# Integrating Transcriptomics and Hormones Dynamics Reveal Seed Germination and Emergence Process in *Polygonatum cyrtonema* Hua

**DOI:** 10.3390/ijms24043792

**Published:** 2023-02-14

**Authors:** Xiaojing Duan, Wu Jiang, Kunjing Wu, Jiadong Chen, Yaping Li, Zhengming Tao

**Affiliations:** 1 Zhejiang Institute of Subtropical Crops, Zhejiang Academy of Agricultural Sciences, Wenzhou 325005, China; 2 Beijing Advanced Innovation Center for Tree Breeding by Molecular Design, College of Forestry, Beijing Forestry University, Beijing 100107, China; 3 Zhejiang Provincial Key Laboratory of Resources Protection and Innovation of Traditional Chinese Medicine, Zhejiang A&F University, Hangzhou 311300, China

**Keywords:** *Polygonatum cyrtonema* Hua, seed germination, seed emergence, hormone, transcriptome

## Abstract

*Polygonatum cyrtonema* Hua is a traditional Chinese herb propagated using rhizomes, and excessive demand for seedlings and quality deterioration caused by rhizome propagation has highlighted that seed propagation may be an ideal solution to address these issues. However, the molecular mechanisms involved in *P. cyrtonema* Hua seed germination and emergence stages are not well understood. Therefore, in the present study, we performed transcriptomics combined with hormone dynamics during different seed germination stages, and 54,178 unigenes with an average length of 1390.38 bp (N50 = 1847 bp) were generated. Significant transcriptomic changes were related to plant hormone signal transduction and the starch and carbohydrate pathways. Genes related to ABA(abscisic acid), IAA(Indole acetic acid), and JA(Jasmonic acid) signaling, were downregulated, whereas genes related to ethylene, BR(brassinolide), CTK(Cytokinin), and SA(salicylic acid) biosynthesis and signaling were activated during the germination process. Interestingly, GA biosynthesis- and signaling-related genes were induced during the germination stage but decreased in the emergence stage. In addition, seed germination significantly upregulated the expression of genes associated with starch and sucrose metabolism. Notably, raffinose biosynthesis-related genes were induced, especially during the emergence stage. In total, 1171 transcription factor (TF) genes were found to be differentially expressed. Our results provide new insights into the mechanisms underlying *P. cyrtonema* Hua seed germination and emergence processes and further research for molecular breeding.

## 1. Introduction

Polygonati rhizoma (“Huangjing” in Chinese), the rhizome of several *Polygonatum* species, including *Polygonatum cyrtonema* in the Liliaceae family, has been extensively used as a traditional medicinal and food material in China for over 2000 years [1]. Polysaccharides, steroidal saponins, flavonoids, and alkaloids are the main active ingredients, and polysaccharides extracted from polygonati rhizoma are advantageous for reducing blood sugar levels, protecting the cardiovascular system, and for anti-aging [2]. Currently, with the increasing importance of the life and health industry, *P. cyrtonema* Hua is now popular in functional foods, such as functional beverages, crisp tablets, and syrup products [3]. This widespread usage of *P. cyrtonema* Hua in traditional Chinese medicines and foods has led to a massive demand for seedlings.

Currently, rhizome propagation (asexual reproduction) and seed propagation are the two conventional methods for obtaining seedlings. However, rhizome propagation tends to cause many problems during practical application. For example, in practice, only rhizomes with buds have the ability to germinate, whereas it is difficult to select and successfully implement this propagation mode in practice; therefore, the practical germination rate by rhizome propagation is relatively low, and rhizome propagation is prone to causing deterioration of the medicinal quality of *P. cyrtonema* Hua. Compared with rhizome propagation, seed propagation can not only avoid quality deterioration but can also promote germplasm improvement and innovation. 

The seed dormancy of *P. cyrtonema* Hua is known to be morphological and physiological dormancy [4]. Many genes are involved in seed germination, and their roles range from encoding transcriptional regulators and sensor complexes to influencing downstream metabolic reactions, including hormones, carbohydrates, and environmental factors [5,6]. Phytohormones, such as auxin [7], gibberellic acid [8], cytokinin [9], and abscisic acid [8,10], regulate seed germination and emergence. During recent years, some studies on the seed germination of *P. cyrtonema* Hua have been published, and Chen et al. (2020) [11] studied endogenous hormone dynamics during seed germination. The results showed that GA_3_ was the key hormone to promote seed germination in *P. cyrtonema* Hua. Liu et al. (2021) mainly shed light on transcriptome and metabolome during seed germination and clarified that phenylpropanoid and flavonoid biosynthesis could be beneficial to the germination [12]. 

However, compared with the model plants on seed dormancy and germination, in addition, it was also observed that the emergence process of *P. cyrtonema* Hua seeds was an obstacle to overcome [4,11], and data on the action mechanisms of comprehensive signaling pathways in *P. cyrtonema* Hua during seed germination and emergence process still need further investigation. Therefore, the main purposes of this study were to explore (1) phytohormonal dynamics in *P. cyrtonema* Hua during seed germination and emergence and (2) key regulatory genes and their functions during *P. cyrtonema* Hua seed germination and emergence. We can offer comprehensive data to characterize crucial genes during *P. cyrtonema* Hua seed germination and emergence progression based on these expression profiles.

## 2. Results

### 2.1. Morphological Changes during Seed Germination Process in P. cyrtonema Hua

**Figure 1:**
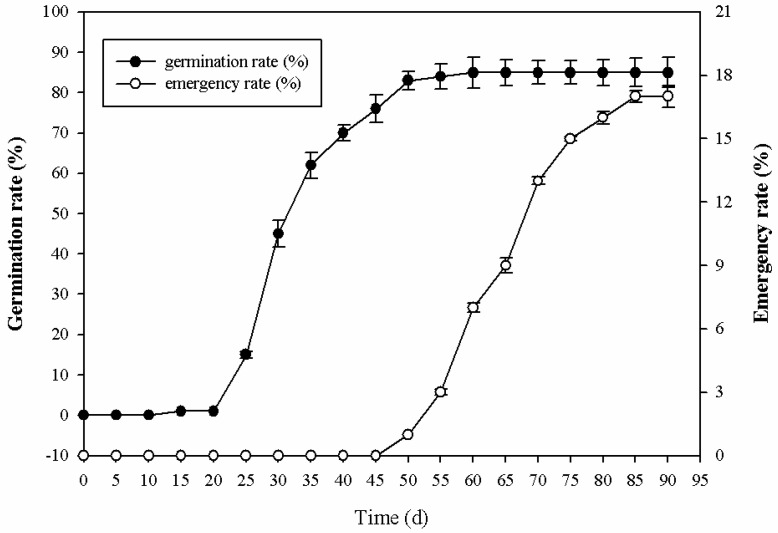
Seed germination rate and emergence rate of *P. cyrtonema* Hua.

The germination and emergence rates of seeds were calculated at 5 d intervals at 25 °C under 16 h daylight in the growth chamber and are shown in Figure 1. Seeds started to germinate 25 d after sowing, the germination rate was 15%, and the germination rate continued to increase sharply until 40 d of sowing (70%) and then slowed down, reaching a maximum rate of 85% after 55 d of sowing. Seedling emergence was much slower than its germination. Seeds started to emerge 50 d after sowing, and the emergence rate continued to increase and reached a maximum after 90 days of sowing (17%). 

### 2.2. Phytohormonal Dynamics of P. cyrtonema Hua during Seed Germination Process

**Figure 2:**
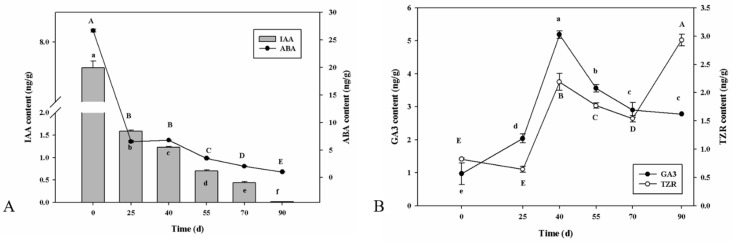
Phytohormonal dynamics content dynamics during seed germination and emergence process in *P. cyrtonema* Hua. Note: Different lowercase and capital letters indicate significant differences (*p* < 0.05) among days of IAA, GA_3_, ABA, and TZR contents, respectively.

Phytohormones play a key role in regulating seed germination. Therefore, the phytohormone content of seeds at specific germination stages was analyzed. Abscisic acid (ABA) content reduced from Day 0 (26.6698 ng/g) to Day 25 (6.5167 ng/g) and continued to decrease during the whole process, reaching a minimum (1.0216 ng/g) after 90 d of sowing (last stage of emergence). The trend of IAA content was similar to that of ABA content. Auxin (IAA) content was highest (7.5697 ng/g) on Day 0 and then continued to decrease until Day 90, reaching the lowest value of 0.50158 ng/g (Figure 2). These results show that ABA and IAA may play negative roles in seed germination and emergence in *P. cyrtonema* Hua.

Studies on the beneficial role of Gibberellin (GA) in the regulation of seed germination have been published [5]. Consistent with previous research, the GA_3_ content rapidly increased from Day 0 (0.9700 ng/g) to Day 40 (maximum 5.1900 ng/g), then decreased, and the minimum content (2.7800 ng/g) was observed after 90 d of sowing (Figure 2). From Day 0 to Day 40, the increase in the dynamics of GA_3_ content was observed from Day 0 to Day 40 but not from Day 55 to Day 90, which indicated that GA_3_ may play a positive role in regulating seed germination. The dynamics of trans-Zeatin Riboside (TZR) content were almost consistent with that of GA_3_ content. TZR content increased sharply until Day 40 (2.1900 ng/g), then dropped to 1.5440 ng/g (after 70 d of sowing, in the middle of the emergence stage), followed by a second rapid increase (2.9300 ng/g), which may imply that TZR functioned not only during seed germination, but also during the late emergence stage of *P. cyrtonema* Hua. 

### 2.3. Transcriptome Analysis and Identification of Differentially Expressed Genes (DEGs) in P. cyrtonema Hua during Seed Germination and Emergence Process

The physiological parameters of seeds during the whole germination process revealed that distinct stages of seed germination and seedling emergence occurred in *P. cyrtonema* Hua. Subsequently, samples of DS (non-germinating stage, Day 0), GS phase (germination stage, Day 40), and the early stage of ES phase (emergence stage, Day 70) were selected for subsequent transcriptome RNA−seq and analysis. Total RNA was extracted from the seeds of the three different sample groups in three biological replicates designated DS−1, DS−2, DS−3; GS−1, GS−2, GS−3; ES−1, ES−2, ES−3, and then sequenced using the Illumina HiSeq Xten platform. More than 93% Q30 bases were acquired (Table 1).

**Table 1:** Summary of generated RNA-seq data.

Sample ID	Base Number	GC Content	% > Q30	Clean Reads	Mapping Rate (%)
DS−1	5,866,481,748	48.87%	94.26%	19,597,195	62.69%
DS−2	6,411,437,502	49.45%	94.34%	21,413,251	65.74%
DS−3	6,471,178,576	49.09%	93.72%	21,619,346	64.35%
GS−1	6,905,162,498	48.20%	94.47%	23,066,585	62.39%
GS−2	7,091,894,902	48.13%	94.11%	23,688,736	62.47%
GS−3	6,177,043,546	48.02%	94.02%	20,633,298	61.89%
ES−1	6,139,693,670	48.68%	94.42%	20,505,088	62.84%
ES−2	6,293,346,204	48.47%	93.95%	21,019,827	62.12%
ES−3	6,307,247,984	48.63%	94.11%	21,065,521	62.98%

A total of 54,178 unigenes were identified. The mean length was 1390.38 bp, and the N50 value was 1847 bp (Table 2).

**Table 2:** Results of transcriptome unigenes.

Total Number	200−500 bp	501−1000 bp	1001−2000 bp	>2000 bp	Total Number	Total Length	N50 Length	Mean Length bp
54,178	7758	16,896	18,175	11,349	54,178	75,328,238	1847	1390.38

**Figure 3:**
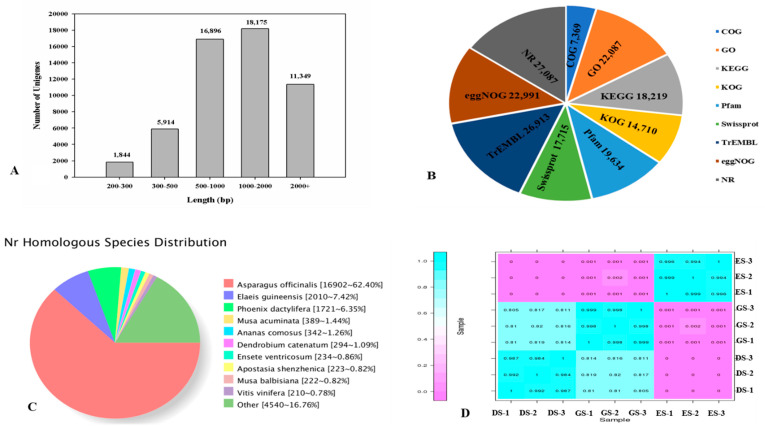
Characteristics of unigenes generated by Illumina sequencing. (**A**) The length distribution of unigenes in *P. cyrtonema* Hua. (**B**) The annotation of unigenes among databases. (**C**) Species distribution of all annotated unigenes. (**D**) Correlation analysis of the samples.

There were 7758 unigenes with lengths < 500 bp, 16,896 unigenes within the length range of 500–1000 bp, 18,175 unigenes within 1000–2000 bp, and 11,349 unigenes with lengths > 2000 bp (Figure 3A). Several complementary methods were used to annotate the unigenes. The assembled unigenes were searched against databases with an E-value of less than 1.0 × 10^−5^. In total, 27,610 (50%) unigenes were annotated in at least one database. Compared with the other seven databases, the NR database showed the highest (27,087) unigenes (Figure 3B). With reference to species, the unigene sequences exhibited maximum similarity to gene sequences from *Asparagus officinalis* (16,902), followed by *Elaeis guineensis* (2010) and *Phoenix dactylifera* (1721, Figure 3C). Correlation analysis revealed a significant correlation among the biological replicates (Figure 3D).

**Figure 4:**
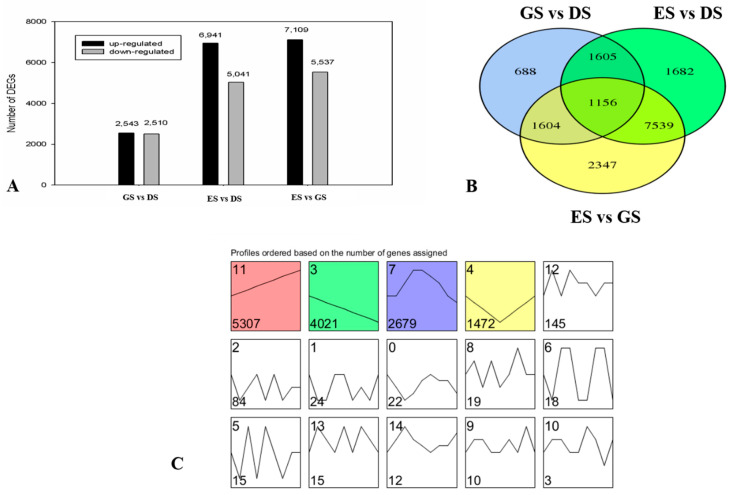
Expression patterns of differentially expressed genes (DEGs) during different stages in *P. cyrtonema* Hua. (**A**) Changes in gene expression profile during different dormancy phases. (**B**) Venn diagram of DEGs. (**C**) Trend analysis of all DEGs. The cluster trend of white background is not significant, and the trend of color background is significant (*p* < 0.05).

Unigenes with *q* value < 0.05 and |log2FC| ≥ 1 were defined as DEGs. Among the DEGs, 2543 and 6941 were upregulated, and 2510 and 5041 genes were downregulated at GS and ES stages, respectively. Compared to GS, 7109 upregulated and 5537 downregulated unigenes were identified at ES. The number of DEGs in ES vs. GS was the highest, followed by ES vs. DS and GS vs. DS (Figure 4A), which indicated that, compared with DS and GS, there was a major difference of ES. To further determine the DEGs associated with seed germination, a Venn diagram was drawn between ES and DS, ES and DS, and ES and ES, and 1156 DEGs were found to intersect all three groups (Figure 4B). Gene expression profile clustering using STEM was performed to further explore the gene expression patterns. The results showed that all DEGs were assigned to 15 different profiles and four profiles were identified as significant (Figure 4C).

**Figure 5:**
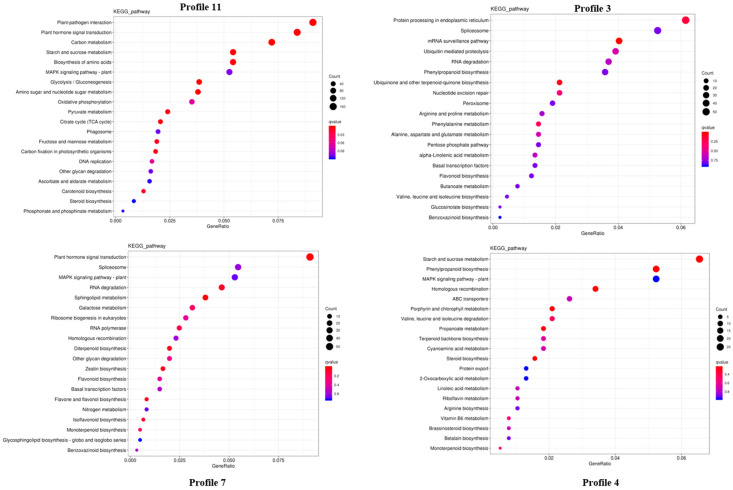
KEGG analysis of significant clusters (*p* < 0.05) in *P. cyrtonema*.

According to the KEGG analysis (Figure 5) of significant clusters, profiles 11 and 4 showed upregulated DEGs, whereas profiles 7 and 4 showed downregulated genes. Plant hormone transduction, starch and sucrose metabolism, and fructose and mannose metabolism pathways were significantly enriched in four profiles, indicating that these pathways played an important role in the germination and emergence processes of *P. cyrtonema* Hua; therefore, DEGs related to these pathways were further analyzed. 

### 2.4. Expression Profiles of Plant Hormones and Signal Transduction Genes in P. cyrtonema Hua during Seed Germination

**Figure 6:**
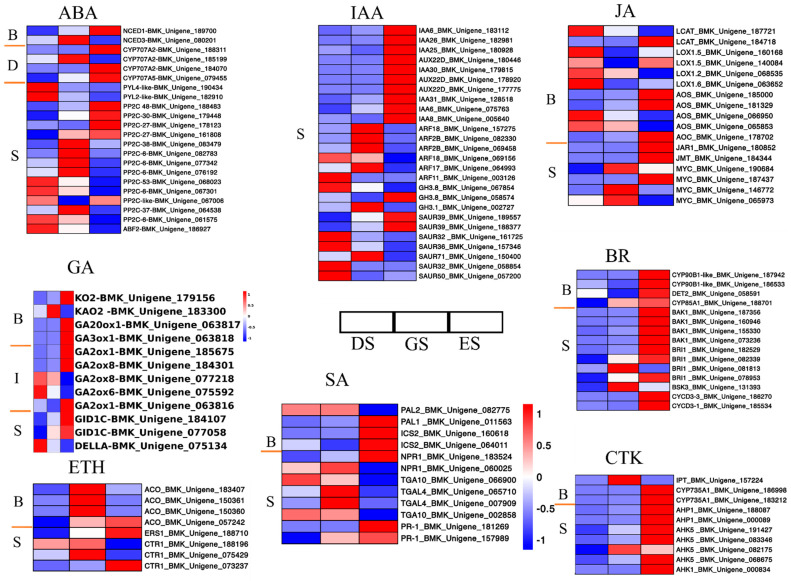
Expression profiles of the DEGs involved in plant hormone biosynthesis, degradation, and signaling pathways in *P. cyrtonema* seeds during germination process. Note: B means Biosynthesis, D means degradation, I means inactivation, and S means signaling.

Based on the KEGG annotation, DEGs related to phytohormone pathways play an important role in seed germination. The two key enzymes that regulate ABA synthesis and catabolism are 9−cis−epoxycarotenoid dioxygenase (NCED) and CYP707A, respectively. In this study, one downregulated DEG encoding NCED and four upregulated DEGs encoding CYP707A were found, which were consistent with ABA content (Figure 2) from DS to ES stages. The ABA signaling pathway comprises three main elements, PYR/PYL/RCAR, type 2C protein phosphatase (PP2C), and SNF1−related protein kinase 2 (SnRK2), which together offer a double-negative regulatory system. In this study, two genes encoding PYL, 13 genes encoding PP2C, and one gene encoding ABF were induced during germination. These results indicate that the ABA signaling pathway is negatively regulated during the seed germination process of *P. cyrtonema* Hua (Figure 6).

In the GA biosynthesis and signal transduction pathway, genes encoding ent−kaurene oxidase (KO), ent−kaurenoic acid oxidase (KAO), gibberellin 20 oxidase (GA20ox), gibberellin 2 oxidase (GA2ox), gibberellin 3 oxidase (GA3ox), GID, and DELLA proteins were found. Overall, DEGs related to GA biosynthesis and signal transduction were upregulated, indicating a positive role for GA in accelerating the seed germination process of *P. cyrtonema* Hua (Figure 6).

In this study, DEGs associated with IAA-mediated signaling displayed a downward trend from DS to ES. Ten auxin−induced protein IAA6 (AUX/IAA) genes were upregulated in ES, six auxin response factors (ARF) were upregulated in GS, two of the three auxin-responsive GH3-like protein (GH3) genes were upregulated, and seven DEGs encoding SAUR were identified, including two upregulated unigenes and five downregulated unigenes (Figure 6).

The expression levels of genes encoding IPP transferase (IPT), Cytochrome P450(CYP735A) involved in cytokinin (CTK) synthesis and histidine−containing phosphotransferase protein (AHP), histidine kinase (AHP) related to CTK signaling gradually increased during seed germination progression (Figure 6). 

DEGs related to ethylene biosynthesis and signaling pathways showed a trend of upregulation during the seed germination process of *P. cyrtonema* Hua (Figure 6). Four DEGs encoding 1−aminocyclopropane-1-carboxylate oxidase (ACO, key enzymes regulating ethylene synthesis) were identified; three were upregulated in GS, and one was upregulated in GS and ES. One gene encoding the ethylene receptor (ERS1) and one encoding the ethylene-responsive protein kinase Le−CTR1(CTR1) were upregulated, and two genes encoding CTR1 showed the opposite trend.

The expression levels of genes associated with Brassinolide (BR), Jasmonic acid (JA), and salicylic acid (SA) biosynthesis and signaling transduction were also analyzed and a series of genes were identified, including those encoding cytochrome P450 enzyme (CYP90B1), Steroid 5−alpha−reductase (DET2), cytochrome P450 (CYP85A1), LRR receptor kinase (BAK1), brassinosteroid insensitive1 (BRI), BR-signaling kinase (BSK) in BR pathway, lecithin-cholesterol acyltransferase-like (LCAT), lipoxygenase (LOX), allene oxide synthase (AOS), allene oxide cyclase (AOC), jasmonic acid amido synthetase (JAR), jasmonate O−methyltransferase (JMT) of JA pathway, phenylalanine ammonia−lyase (PAL), isochorismate synthase (IS), NPR1, TGA, and pathogenesis−related protein (PR−1) in the SA pathway, that were differentially expressed during seed germination (Figure 6).

### 2.5. Expression Profiles of Carbohydrate Metabolism in P. cyrtonema Hua during Seed Germination and Emergence Processes

**Figure 7:**
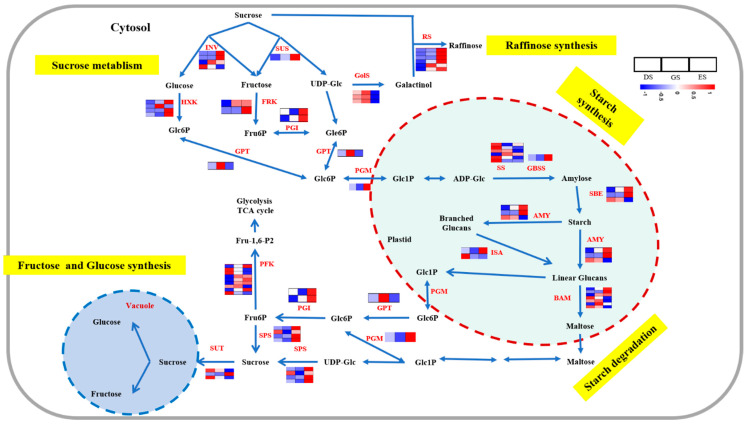
Expression profiles of DEGs involved in carbohydrate metabolism pathways in *P. cyrtonema* seeds during the germination process.

According to STEM analysis and KEGG annotation, DEGs related to carbohydrate function in seed dormancy transition; hence, we analyzed the expression patterns of 54 DEGs related to carbohydrates. Starch is the main source of sugar in plants. In this study, the genes involved in starch synthesis, including starch synthase (SS) and granule-bound starch synthase (GBSS), were downregulated, whereas the genes involved in starch degradation, including starch branching enzyme (SEB), α-amylase (AMY), and β-amylase (BMY), showed a significant increase from DS to ES stage. Similar to genes involved in starch degradation, genes belonging to fructose and glucose, raffinose synthesis such as phosphoglucomutase (PGM), sucrose phosphate synthase (SPS), sucrose transport protein (SUT), glucose−6−phosphate/phosphate translocator protein (GPT), phosphofructokinase (PFK), fructokinase (FPK), hexokinase (HXK), galactinol synthase (GolS), and raffinose synthase (RS) were upregulated during the seed dormancy release process (Figure 7). Detailed information of DEGs involved in carbohydrate metabolism is shown in Figure S1 (Supplementary Files).

### 2.6. TFs Were Differentially Expressed in P. cyrtonema Hua during Seed Germination and Emergence Process

**Figure 8:**
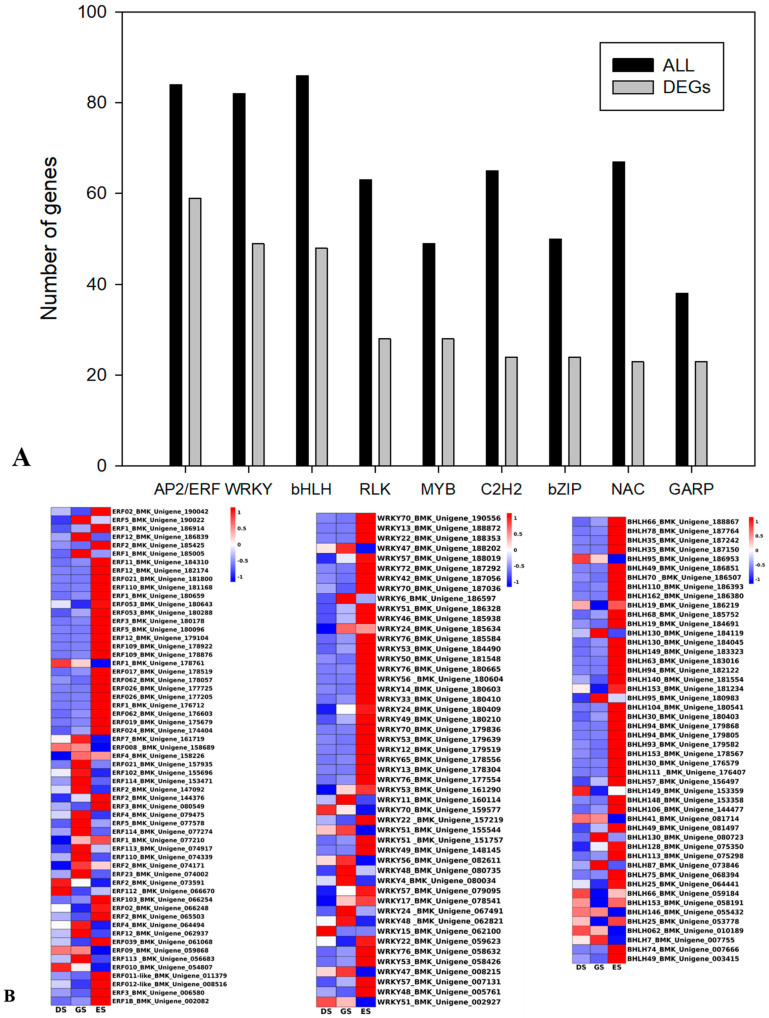

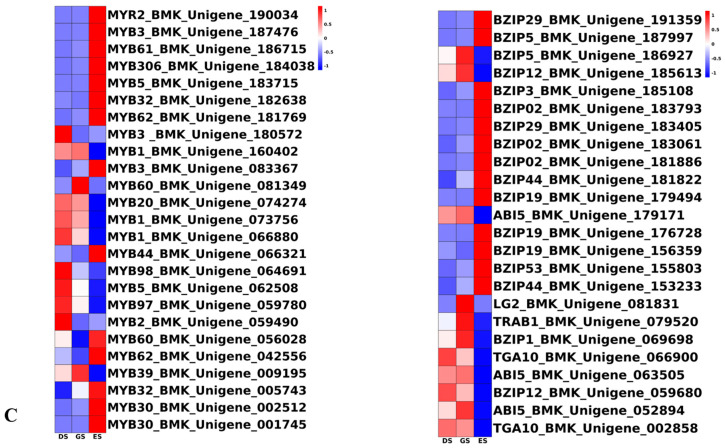
Number of differentially expressed transcription factors during seed germination in *P. cyrtonema.* (**A**) Top TF families according to gene number. (**B**) DEGs heatmap of ERFs, WRKYs, and BHLHs. (**C**) DEGs heatmap of MYBs and BZIPs during different germination phases in *P. cyrtonema* seeds.

In total, 1171 TF genes (747 up−regulated and 424 down-regulated) that were active during seed germination were identified. These TFs were mainly AP2/ERF (59), WRKY (49), bHLH (48), RLK (28), MYB (25), C2H2 (24), bZIP (24), NAC (23), and GARP (23) (Figure 8A). Among them, most showed an up-regulated trend from DS to ES, and it is worth noting that gene expression in ES was significantly different from the other two stages, which that indicated molecular mechanisms in ES were worth studying. In addition, compared with DS and GS, DEGs belonged to MYBs and BZIPS tend to be down-regulated in ES. For example, BMK_Unigene_066900 and BMK_Unigene_002858 encoded a BZIP transcription factor TGA10, TGA10 functioned in seed dormancy control process, showing a significant decrease in ES. We also identified the homologs of ABI5 (abscisic acid-insensitive 5, belong to BZIPs, gene ID: BMK_Unigene_179171, BMK_Unigene_063505, BMK_Unigene_052894, respectively), which play a vital role in versatile pathways regarding seed dormancy and germination [13]; Figure 8B,C). From these results, it was speculated that various differentially expressed TFs act as potential regulators of biological processes responsible for the dormancy release progression of *P. cyrtonema* seeds.

### 2.7. Validation of RNA-Seq by qRT-PCR Analysis

**Figure 9:**
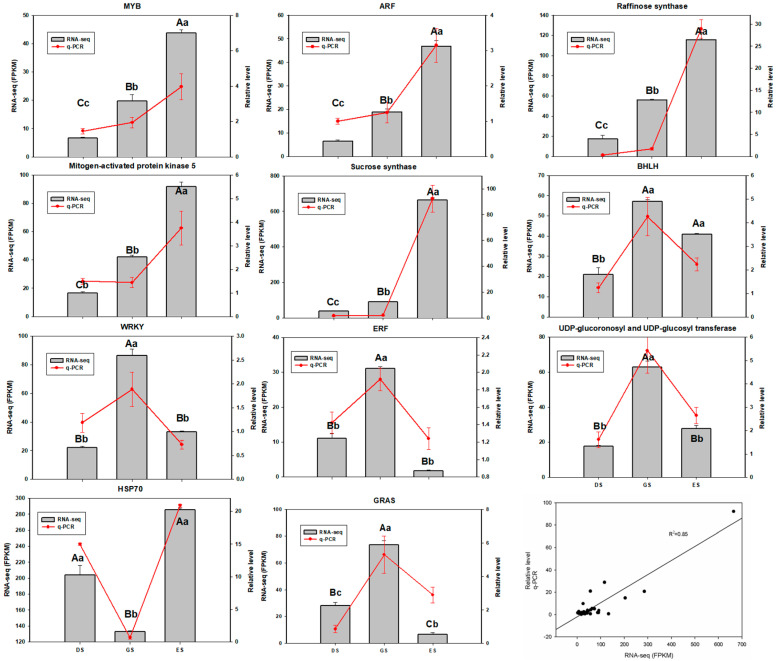
The expression of 11 DEGs and comparison between qRT−PCR and RNA−seq. Note: Different capital letters indicate significant differences among different stages of RNA-seq; different lowercase letters indicate significant differences among different stages of qRT−PCR.

Eleven candidate unigenes were randomly selected to confirm the reliability of the RNA-seq using qRT−PCR. The qRT−PCR results correlated well with the RNA−seq data, indicating the reliability and accuracy of RNA−seq (Figure 9).

## 3. Discussion

*P. cyrtonema* Hua is widely used in functional foods because of its beneficial effects on human health [3]. The widespread use of *P. cyrtonema* Hua in traditional Chinese medicines and foods has resulted in an enormous demand for its seedlings. Seed propagation not only prevents quality deterioration but can also promote germplasm improvement and innovation. However, the molecular mechanisms underlying *P. cyrtonema* Hua seed germination and emergence are still poorly understood. Therefore, we recorded the seed germination course and selected the main development stages, carrying out transcriptome sequencing to shed light on the molecular mechanism of seed germination and emergence in *P. cyrtonema* Hua.

### 3.1. Regulation of Plant Hormones in P. cyrtonema Hua during Seed Germination and Emergence Progression

In recent decades, there has been an extensive review of the role of hormone homeostasis in controlling seed dormancy and germination [14]. ABA levels are closely related to seed dormancy status. In this study, ABA levels showed a significant decline during the germination process (Figure 2), which was similar to that of red bayberry [15], celery [16], ginkgo [17], and pear [18]. Concomitantly, the expression profiles of multiple ABA biosynthesis and deactivation genes, including NCEDs and CYP707As, were highly consistent with the dynamics of ABA (Figure 2). NCED, the main enzyme involved in ABA biosynthesis, converts 9−cis−epoxycarotenoids into C15. CRY707A, a key ABA catabolism gene, regulates ABA levels by converting ABA into 8′-hydroxy ABA [19]. Four CYP707A genes (AtCYP707A1 to AtCP707A4) regulate ABA levels in *Arabidopsis thaliana* [20]. In this study, one NCED gene showed an increase and one CYP707A gene decreased, while one NCED and three CYP707A genes showed decreasing and increasing trends, respectively, during seed germination. ABA biosynthesis and catabolism were activated during seed germination, and ABA catabolism was more activated. ABA content combined with up-regulation of CYP707A indicated that PcCYP707As may participate in regulating the seed germination progression of *P. cyrtonema* Hua (Figure 6). In addition, ABA signal transduction and regulation also influence seed germination [21,22]. PP2C proteins act as negative regulators in the ABA signaling pathway [23]. In this study, expression profiles of two PYL and 13 PP2C genes changed significantly during seed germination process, and two PYL genes and nine of the 13 PP2C genes showed a down-regulation trend from DS to ES stage (Figure 6), which may have contributed to *P. cyrtonema* Hua seed germination (Figure 6). The specific roles of other PP2C genes require further research (Figure 6). These findings imply that the ABA signaling pathway negatively participates in seed germination in *P. cyrtonema* Hua.

GA is a major hormone that promotes the germination of seeds. In this study, GA_3_ (an important active gibberellin) concentration steadily increased from day 0 to day 40, and then decreased in the ES stage (Figure 2). The expression profiles of some GA biosynthesis genes, including KO, KAO, GA3oxs, and GA20oxs, showed a highly similar trend to the changes in GA_3_ levels synchronously (Figure 2). *OsGA3oxs* and *OsGA20oxs* were highly expressed in the embryo during the germination of rice seeds, and in this study, GA3oxs and GA20oxs-related genes increased from DS to ES, which is consistent with previous research [5]. GA regulates signaling via GID1-DELLA-SCFSLY1/GID2 [24]. In *P. cyrtonema* Hua seeds, two GID1 genes exhibited high expression levels from DS to ES, and one gene encoding the DELLA protein showed the opposite trend (Figure 6). The DELLA protein acts as a negative regulator of the GA signaling pathway [25]. These results provide evidence that GA may play a positive role in the release of seed germination in *P. cyrtonema* Hua.

The specific role of IAA in seed germination may vary between species. Some studies have reported that IAA levels increase during seed imbibition and after ripening in *Arabidopsis*, pear [18], and wheat [26]; on the contrary, more studies have implied that IAA acts through interaction with ABA and thereby inhibits seed germination and pre-harvest sprouting instead of promoting seed dormancy [26]. In the present study, the trend in IAA content was similar to that of ABA content, which decreased from DS to ES. Aux/IAA is an extremely important inhibitory component that forms heterodimers with the transcription factor ARF, which is responsible for regulating gene expression in the auxin signaling pathway and inhibiting the transcriptional regulatory activity of ARF. When auxin binds to the receptor TIR1, the binding of SCF and Aux/IAA is enhanced, Aux/IAA protein degradation is promoted, and ARF is released. Aux/IAA is also downstream of ARF, which is rapidly induced by auxin, forming a negative feedback loop in the auxin signaling pathway [27]. Consistent with IAA content, genes encoding Aux/IAA were upregulated, and genes related to ARF, GH3, and SAUR were downregulated (Figure 8), which indicates that auxin may negatively regulate the seed germination of *P. cyrtonema* Hua.

Ethylene also intervenes in seed dormancy. CTR1 is a key negative regulator of ethylene signaling; however, this repression is released in the presence of ethylene [28]. CTR1 does not promote seed germination through a typical ethylene signal but functions by negatively regulating the ABA signal. Studies have reported that the germination of the negative regulator CRT1 mutant is significantly accelerated [29]. In this study, four DEGs encoding ACO, key enzymes regulating ethylene synthesis, were identified; three were upregulated in GS, one was upregulated in GS and ES, and two CTR1 genes declined during seed germination and emergence processes (Figure 8), indicating that ethylene may positively participate in the seed germination process in *P. cyrtonema* Hua.

BR reduces the dormancy effect of ABA on seeds to promote germ growth [30]. DET is the main enzyme in BR biosynthesis, but it has been reported that ABA in Arabidopsis has a significant inhibitory effect on the germination of BR synthetic mutants det2−1 and BRI mutants [31]. In addition, BRI1, a key transcription factor in the BR signaling pathway, has also been shown to interact directly with ABI 5 and interfere with its transcriptional activity [32]. In the present study, some BR pathway-related genes, including DET, BSK1, and BRI, were differentially expressed during seed germination and emergence, indicating that BR may play a positive role in *P. cyrtonema* Hua seed germination. 

ABA induced by exogenous addition or stress usually induces the biosynthesis of JA; therefore, it is generally believed that JA and ABA can coordinate and control most biological reactions, such as plant seed germination [33]. Our study identified 17 differentially expressed genes involved in the synthesis, catabolism, and signaling of JA, and most DEGs showed a downward trend during seed germination, indicating that JA might negatively participate in the seed germination of *P. cyrtonema* Hua (Figure 2). These findings were similar to those observed in wheat seeds [34].

CTK has also been reported to regulate seed dormancy through interactions with ABA/GA levels and signals [21]. CTK positively regulates seed germination in dicot species and functions by intervening in the ABA pathway [35]. Ten DEGs annotated as CTK biosynthesis and metabolic and signaling genes were found (Figure 8) and showed an increasing trend, mainly in the ES stage (Figure 2), which was consistent with the TZR content, implying that CTK may have a positive role in the seed emergence process. The role of SA in seed dormancy release has barely been studied; however, based on our results, 12 DEGs annotated as SA biosynthesis and metabolic and signaling genes were discovered with distinct expression trends (Figure 8), which signified that SA may function in seed germination, and the specific function needs further investigation.

### 3.2. Regulation of Carbohydrate in P. cyrtonema Hua during Seed Germination and Emergence Progression

It has been reported that starch degradation and sugar accumulation are elevated during the transition seed germination and emergence and might be vital for promoting seed dormancy release [36]. The degradation of starch during seed germination is an important biochemical mechanism that increases water retention and osmotic potential in cells and provides energy for seed germination [37]. In this study, based on KEGG analysis, starch and sucrose metabolism were significantly enriched. Numerous genes related to starch biosynthesis and degradation have been identified. Starch synthase, GBSS, and SBE are three key enzymes involved in starch biosynthesis [38]. In this study, six starch synthase genes and one SBE gene were downregulated, while one gene encoding GBSS and two genes encoding SBE showed an increasing trend, especially in ES stage, whereas three genes encoding beta-amylase and two genes encoding α-amylase, which were associated with the breakdown of starch, showed upregulation (Figure 7). Sucrose phosphate synthase is an important enzyme that participates in sucrose metabolism and biosynthesis [39]. Four sucrose phosphate synthase genes were also induced during seed germination emergence, indicating possible sucrose biosynthesis (Figure 7). RS and GolS are two key enzymes in raffinose synthesis [40], in this study, three GolS genes were induced in GS, while six RS genes were induced in ES, suggesting that raffinose functions in whole seed germination and emergence of *P. cyrtonema* Hua. In addition, the genes involved in hexogenesis pathway, including (INV, SUS, HXK, FRK, and PGI), also increased during the seed germination process (Figure 7). Moreover, carbohydrates participate in crosstalk with hormones to regulate seed germination and dormancy. AMY gene expression is activated by GA through GA response element (GARC) [41]. In this study, nine genes encoding GA biosynthesis and signals and two genes encoding AMY increased during seed germination, which may provide indirect evidence to support this conclusion. 

### 3.3. Transcription Factors Play a Pivotal Role in P. cyrtonema Hua Seed Germination and Emergence Progression

TFs mediate the seed germination process by interacting with hormones and carbohydrates; therefore, we also identified TF genes that are differentially expressed during the seed germination process. Consistent with a previous study [21], the ERF, WRKY, bHLH, MYB, bZIP, and NAC genes were identified (Figure 8). MYB promotes seed dormancy by regulating the transcription of genes related to ABI4 and ABA synthesis. During seed dormancy, WRKY binds to the promoter of the key target gene RAV1 to inhibit its expression and regulate ABA content, whereas WRKY further inhibits the GA-inducible α-amylase gene [42]. ARF10/6 regulates the expression of ABI3, maintains the intensity of the ABA signal, and promotes seed dormancy; GARC includes two key elements, GARE and TA box [42]. GAMYB is a key transcription factor induced by GA, which directly binds to GARE and induces the expression of a series of hydrolase genes, including amylase gene [43]. The TA box is known as the sugar response element (SRE) in the sugar reaction complex. MYB is induced by sugar starvation, followed by its interaction with the TA box to promote seed germination. These findings imply that ERF, WRKY, and BZIP are important components of the regulatory network that mediates seed germination in *P. cyrtonema* Hua.

## 4. Materials and Methods

### 4.1. Plant Materials and Treatments

The seeds were harvested in September from Qingyuan County, Lishui City, Zhejiang Province (27°37′ N, 119°3′ E). The seeds were washed with distilled water, soaked with 5% sodium hypochlorite for 20 min, then washed with distilled water four to five times to remove the residual liquid on the surface of the seeds, and finally sowed in 5 × 10 nursery plate (2 grains per hole, 100 grains in each plate as per replicate, three replicates). One sample included 20 seeds. In total, 60 seed were obtained from three plates. Germination rate and emergence rate were measured every 5 days. Samples (each sample included 10 seeds) were collected randomly at different stages and rapidly frozen in liquid nitrogen and then stored at −80 °C until subsequent experiment and analysis. The germination of seeds was deemed as if the radicle broke through the seed coat, and the emergence was as if the first cotyledon was unfolded. There was water every 2–3 days, and necrotic seeds were removed on time.

### 4.2. Measurements of Hormone Contents

A mixture of 100 mg of sample and 1 mL of extract (acetonitrile:water = 1:1) was extracted on ice for 4 h. The supernatant was obtained by centrifugation for ten minutes under condition of 4 °C and 12,000× *g*, then 35 mg of C18 filler was added. It was shaken violently for 30 s, centrifuged under 10,000× *g* for 5 min, and the supernatant was obtained; it was dried with nitrogen, dissolved in 200 μL methanol, filtered with 0.22 μM organic phase filter membrane, and put into a refrigerator at −20 ℃ for testing. Hormonal quantification was determined by HPLC-MS/MS (Agilent 1260, Santa Clara, CA, USA) with an Agilent poroshell 120 SB-C18 column (150 mm × 2.1 mm, 2.7 µm), at 30 °C, mobile phase is A:B = (methanol/0.1% formic acid):(water/0.1% formic acid). The gradient parameters of HPLC are shown in Table 3. Selected reaction monitoring conditions are shown in Table 4. 

### 4.3. RNA Extraction and Transcriptome Sequencing

Total RNA was extracted from seeds at DS (dormancy stage), GS (germination stage), and ES (emergence stage) using RNA TRIzol (Tiangen, Beijing, China). RNA integrity was verified by the RNA-free agarose gel electrophoresis and the concentration was measured by 2100 Bioanalyzer (Agilent, Santa Clara, CA, USA). 2 μg RNA per sample was used to construct sequencing libraries according to manufacturer instructions (NEB, Ipswich, MA, USA). All nine libraries were sequenced on the Illumina Hiseq Xten platform (Illumina, San Diego, CA, USA), which was conducted by the Biomarker Technologies Company in Beijing, China. The raw data were uploaded in the National Center for Biotechnology Information (NCBI) BioProject database (accession number PRJNA874511). By removing reads containing adapters, clean data were obtained. Following that, the clean reads were mapped to the transcriptome sequence by HISAT2 tools software. Gene function was annotated based on NR, Nt, Pfam, COG, Swiss−Prot, KOG, KEGG, and GO. Fragments per kilobase of transcript per million fragments mapped (FPKM) were used to normalize gene expression by Cufflinks (version 2.2.2) software [44].

### 4.4. Differential Expression Analysis

With the DESeq R package, differential expression analysis was performed among samples (v1.10.1) [45]. The differential expression of genes (DEGs) was defined as significant when the corrected *p*-value was paired with the false discovery rate—the (q-value) 0.05. Heatmaps were generated using the pheatmap (v1.0.12) of the R package and TBtools [46]. Using the online Short Time-series Expression Miner (STEM) program, we further clustered and compared the gene expression patterns of DEGs during the entire germination stages.

### 4.5. Quantitative Real-Time PCR Validation Statistical Analysis

The results of RNA−seq were validated using qRT−PCR, and qRT−PCR analysis was conducted, including three biological replicates for each sample. 18S was used as the house−keeping gene, and the fold change was calculated using the 2^−∆∆CT^ method. Primer sequences are shown in Table 5.

The data were analyzed using one-way analysis of variance, followed by least significant difference test, and *p* value < 0.05 was considered significant. Graphs were constructed using SigmaPlot version 10 (Systat Software, San Jose, CA, USA) and R Project (R Foundation for Statistical Computing, Vienna, Austria). All data were analyzed using SPSS Statistics version 20 (IBM, Armonk, NY, USA).

## 5. Conclusions

In this study, seed germination and emergence processes in *P. cyrtonema* Hua were uncovered by physiological and transcriptomic analyses. Overall, genes related to ABA, IAA, and JA signaling transduction negatively regulated seed germination and emergence, whereas GA, ethylene, CTK, BR, and SA were positively regulated in seed germination. Notably, CTK played a major role in accelerating emergence instead of GA. In addition, genes related to carbohydrate metabolism, including sucrose, fructose, and raffinose synthase, were also induced for providing the necessary energy during seed germination and emergence. Furthermore, TF genes such as ERF and BZIP showed dynamic expression patterns, which were considered crucial during *P. cyrtonema* Hua seed germination and emergence. Our findings provide transcriptome information on the regulation of seed germination in *P. cyrtonema* Hua.

## Figures and Tables

**Table 3:** Gradient parameters of HPLC.

Time (min)	Velocity of Flow (mL/min)	A%
0–1	0.3	20
1–3	0.3	Increase from 20 to 50
3–9	0.3	Increase from 50 to 80
9–10.5	0.3	80
10.5–10.6	0.3	Decrease from 80 to 20
10.6–13.5	0.3	20

**Table 4:** Selected reaction monitoring conditions for protonated or deprotonated plant hormones ([M+H]+ or [M-H]-).

Hormone	Polarity	Parent Ion (*m*/*z*)	Daughter (*m*/*z*)	De-Clustering Voltage (V)	Collision Energy (V)
GA_3_	−	345.2	143.0 */239.0	−80	−30/−33
ABA	−	263.1	153.1 */204.2	−60	−14/−27
TZR	+	352.2	220.1 */136/202.1	90	25/40/32
IAA	+	176.1	130.1 */102.9	65	12/42

Note: The ions marked with * are quantitative ions.

**Table 5:** Primer sequences.

Gene Id	Primer Name	Sequence (5′ to 3′)	Annotation
Reference gene	18S-F	CAACCATAAACGATGCCGA	18S
Reference gene	18S-R	AGCCTTGCGACCATACTCC	
BMK_Unigene_187871	BMK_Unigene_187871-F	CATACTGGTTTTCTTCCCCTTTT	MYB
BMK_Unigene_187871	BMK_Unigene_187871-R	TTTCAGCTAAGGAAACAACAGG	
BMK_Unigene_187926	BMK_Unigene_187926-F	GTGGCACTAACAAAGGTGAAAGA	ARF 9-like
BMK_Unigene_187926	BMK_Unigene_187926-R	TTCCTCTAATAATTCCTTGCGTGC	
BMK_Unigene_068480	BMK_Unigene_068480-F	GAAGTAATCAACCAAGCAGTC	Raffinose synthase
BMK_Unigene_068480	BMK_Unigene_068480-R	CATCCAAACCAGTCCAGAA	
BMK_Unigene_068480	BMK_Unigene_175000-F	AAAGGGAAATGACCAGAC	MAPK
BMK_Unigene_068480	BMK_Unigene_175000-R	TCCTCCAGTACAACATCT	
BMK_Unigene_185451	BMK_Unigene_185451-F	CGCTCAGGCTTTGTATTCTAT	Sucrose synthase
BMK_Unigene_185451	BMK_Unigene_185451-R	GTATCCGTTGCTTAACTTCCT	
BMK_Unigene_190684	BMK_Unigene_190684-F	TGTAGATGTTGAGGCTGATG	bHLH-MYC
BMK_Unigene_190684	BMK_Unigene_190684-R	TTGTATTACCTTCGCACACT	
BMK_Unigene_067491	BMK_Unigene_067491-F	GCATTCGCTGAAGTCTCTAA	WRKY
BMK_Unigene_067491	BMK_Unigene_067491-R	TCTCCTCTTCTCTTGACCAA	
BMK_Unigene_075429	BMK_Unigene_075429-F	TCTCCTTGAATCCGACACT	ERF
BMK_Unigene_075429	BMK_Unigene_075429-R	GCTACCCACCTCACTATTTAC	
BMK_Unigene_079332	BMK_Unigene_079332-F	GCCCCTTTAACCTTTTGTTG	Zeatin biosynthesis
BMK_Unigene_079332	BMK_Unigene_079332-R	TTGTAGTATCCGCTTATCACG	
BMK_Unigene_081582	BMK_Unigene_081582-F	ATTGACGGTGGAAACAAC	MYB
BMK_Unigene_081582	BMK_Unigene_081582-R	GAAAACGAGAACAGGAGAG	
BMK_Unigene_069145	BMK_Unigene_069145-F	GATGATGAACACGCTGAAGA	HSP70
BMK_Unigene_069145	BMK_Unigene_069145-R	TCTCTGAACTTGCTGAACAC	
BMK_Unigene_003009	BMK_Unigene_003009-F	TCATCATTCTCTCACTGCTTAGTT	GRAS
BMK_Unigene_003009	BMK_Unigene_003009-R	ACCAGATACCTGAAGGAAACAAT	

## Data Availability

The RNA-seq datasets generated during the current study have been uploaded in the National Center for Biotechnology Information (NCBI) BioProject database (accession number PRJNA874511). The other data are included in supplementary information files.

## Supplementary Materials

The following supporting information can be downloaded at: https://www.mdpi.com/article/10.3390/ijms24043792/s1.
