# Supplementary material for: Integrating Transcriptomics and Hormones Dynamics Reveal Seed Germination and Emergence Process in Polygonatum cyrtonema Hua
Source: Int J Mol Sci. 2023 Feb 14;24(4):3792. doi: 10.3390/ijms24043792 (PMC9967326; doi:10.3390/ijms24043792)
Supplement: Supplementary file 1 [file ijms-24-03792-s001.zip › ijms-2138648-supplementary.pdf]

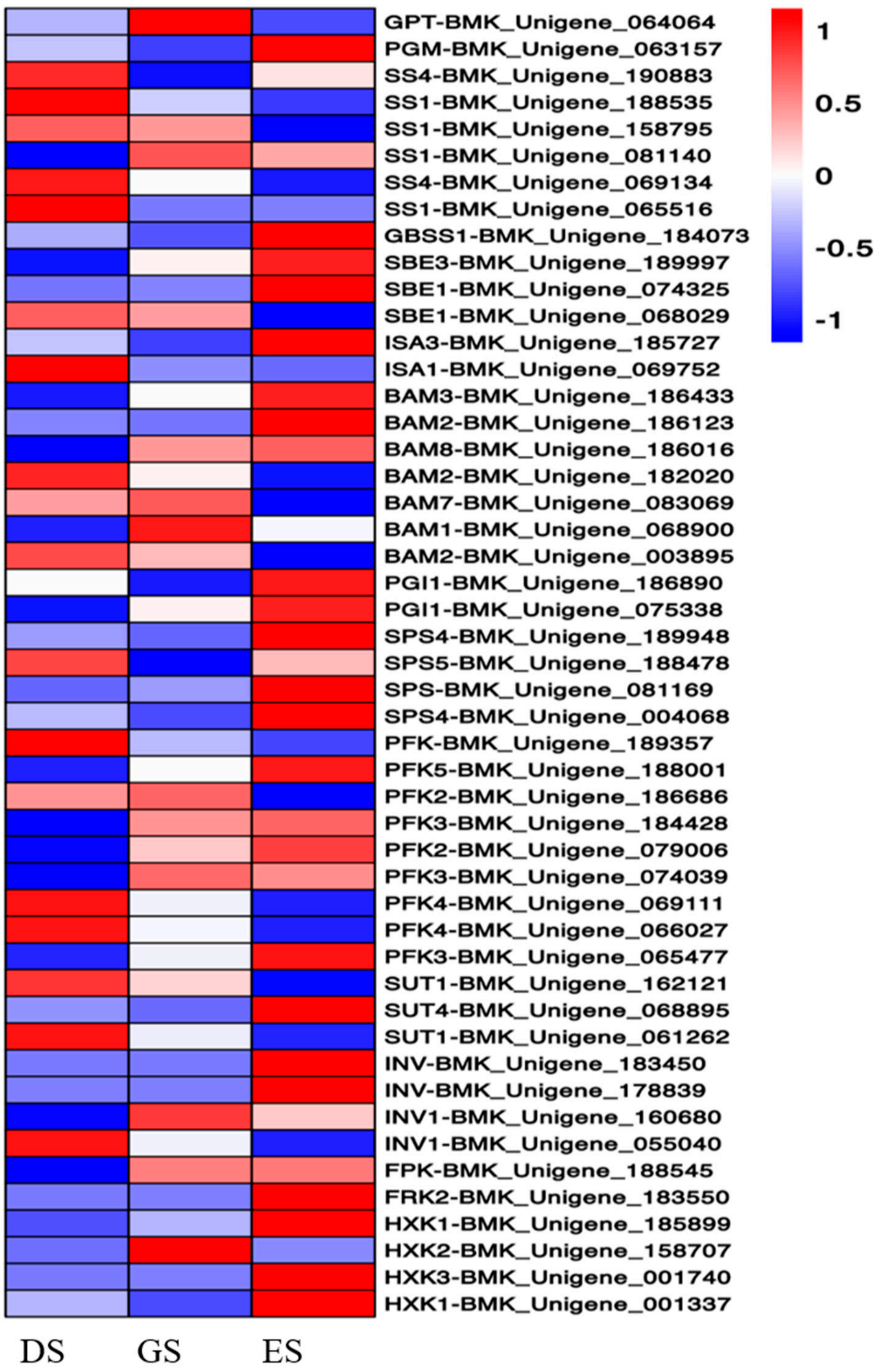

Figure S1: Heatmaps of DEGs involved in Carbohydrate metabolism pathways in *P. cyrtonema* Hua seeds during germination process.

Note: DS means non-germinating stage, GS phase means germination stage, and ES means emergence stage.
